# Supplementary material for: IPI score as a new prognostic index in extensive stage small cell lung cancer
Source: PeerJ. 2025 Nov 10;13:e20343. doi: 10.7717/peerj.20343 (PMC12614095; doi:10.7717/peerj.20343)
Supplement: Supplemental Information 2 [file peerj-13-20343-s002.docx]

Codebook for RAW data:

Sex: 1-Male; 2-Female

Smoker: 1-active; 2-non; 3-ex; 4-secondhand

Hypertension: 0-absent; 1-present

Diabetes mellitus: 0-absent; 1-present

Coronary artery disease: 0-absent; 1-present

Chronic kidney disease: 0-absent; 1-present

Eastern cooperative oncology group scale: 1-ECOG 0-1; 2-ECOG 2; 3-ECOG 3 -4

Liver metastasis: 0-absent; 1-present

Lung metastasis: 0-absent; 1-present

Bone metastasis: 0-absent; 1-present

Brain metastasis: 0-absent; 1-present

Other metastasis: 0-absent; 1-present

Chemoradiotherapy status: 0-absent; 1-present

Number of treatment lines: 0-absent; 1-present

Metastatic 1st-line treatment: 1-platin-etoposid; 2-platin-etoposid-atezolizumab; 3-others

Best response (1 st-line) Best response (1 st-line): 1-stable; 2-progressive; 3-complete; 4- partial

Metastatic 2 nd-line treatment: 1-temozolamide-irinotecan; 2-gemcitabin; 3-vinorelbine; 4-paclitaxel; 5-docetaxel; 6- topotecan; 7-temozolamide; 8-irinotecan; 9-others

Best response (2 nd-line): 1-stable; 2-progressive; 3-complete; 4- partial

Metastatic 3 rd-line treatment: 1-temozolamide-irinotecan; 2-gemcitabin; 3-vinorelbine; 4-paclitaxel; 5-docetaxel; 6- topotecan; 7-temozolamide; 8-irinotecan; 9-others

Best response (3 rd-line): 1-stable; 2-progressive; 3-complete; 4- partial

Exitus: 0-absent; 1-present
