# Supplementary material for: IPI score as a new prognostic index in extensive stage small cell lung cancer
Source: PeerJ. 2025 Nov 10;13:e20343. doi: 10.7717/peerj.20343 (PMC12614095; doi:10.7717/peerj.20343)
Supplement: Supplemental Information 3 [file peerj-13-20343-s003.docx]

STROBE Statement—checklist of items that should be included in reports of observational studies

|  | Item No. | Recommendation | Page  No. | Relevant text from manuscript |
| --- | --- | --- | --- | --- |
| **Title and abstract** | 1 | (*a*) Indicate the study’s design with a commonly used term in the title or the abstract | Page1 | A retrospective cohort study evaluating the prognostic value of the Inflammatory Prognostic Index (IPI) in extensive-stage small cell lung cancer (ES-SCLC) |
|  |  | (*b*) Provide in the abstract an informative and balanced summary of what was done and what was found | Page2 | The study retrospectively analyzed 94 ES-SCLC patients to evaluate the association between IPI score and overall survival (OS). High IPI scores were significantly associated with poorer OS. Multivariate Cox regression confirmed IPI as an independent prognostic factor. |
| Introduction | | | |  |
| Background/rationale | 2 | Explain the scientific background and rationale for the investigation being reported | Page2-3 | While prognosis in ES-SCLC is poor, inflammatory markers have emerged as potential indicators of survival. IPI combines CRP, NLR, and albumin, and may offer a robust prognostic tool.  adequately identified in clinical practice. |
| Objectives | 3 | State specific objectives, including any prespecified hypotheses | Page3 | To evaluate the prognostic significance of IPI in patients with ES-SCLC and determine its relationship with overall survival. |
| Methods | | | |  |
| Study design | 4 | Present key elements of study design early in the paper | Page 3-4 | Retrospective cohort study conducted at Celal Bayar University Hospital between Jan 2012–Nov 2024. |
| Setting | 5 | Describe the setting, locations, and relevant dates, including periods of recruitment, exposure, follow-up, and data collection | Page 4 | Data collected from patient records, including baseline labs and follow-up outcomes. Patients treated with chemotherapy and/or immunotherapy. |
| Participants | 6 | (*a*) *Cohort study*—Give the eligibility criteria, and the sources and methods of selection of participants. Describe methods of follow-up  *Case-control study*—Give the eligibility criteria, and the sources and methods of case ascertainment and control selection. Give the rationale for the choice of cases and controls  *Cross-sectional study*—Give the eligibility criteria, and the sources and methods of selection of participants | Page3 | Inclusion: Adults ≥18 years, confirmed ES-SCLC, complete clinical/lab data.  Exclusion: Secondary malignancy, autoimmune/inflammatory disease, hematologic conditions, infections.  Final sample: 94 patients. |
|  |  | (*b*) *Cohort study*—For matched studies, give matching criteria and number of exposed and unexposed  *Case-control study*—For matched studies, give matching criteria and the number of controls per case |  |  |
| Variables | 7 | Clearly define all outcomes, exposures, predictors, potential confounders, and effect modifiers. Give diagnostic criteria, if applicable | Page 4-5 | Exposure: IPI = (CRP × NLR) / Albumin  Outcome: Overall survival (OS)  Confounders: Age, sex, ECOG-PS, BMI, metastases, comorbidities |
| Data sources/ measurement | 8* | For each variable of interest, give sources of data and details of methods of assessment (measurement). Describe comparability of assessment methods if there is more than one group | Page 4-5 | Labs obtained within 1 week before diagnosis.  Statistical analysis via SPSS 15.0; ROC used to define IPI cutoff (1.25). |
| Bias | 9 | Describe any efforts to address potential sources of bias | Page 4-5 | Exclusion criteria controlled for confounders. Multivariate analysis adjusted for clinical variables. |
| Study size | 10 | Explain how the study size was arrived at | Page 4 | 122 initially reviewed; 28 excluded; 94 analyzed. |

Continued on next page

| Quantitative variables | 11 | Explain how quantitative variables were handled in the analyses. If applicable, describe which groupings were chosen and why | Page 5 | IPI score categorized into "Low" (<1.25) and "High" (≥1.25) using ROC. |
| --- | --- | --- | --- | --- |
| Statistical methods | 12 | (*a*) Describe all statistical methods, including those used to control for confounding | Page 5 | Kaplan-Meier, log-rank, Cox regression for survival.  Multivariate model included significant univariate predictors. |
|  |  | (*b*) Describe any methods used to examine subgroups and interactions |  |  |
|  |  | (*c*) Explain how missing data were addressed |  |  |
|  |  | (*d*) *Cohort study*—If applicable, explain how loss to follow-up was addressed  *Case-control study*—If applicable, explain how matching of cases and controls was addressed  *Cross-sectional study*—If applicable, describe analytical methods taking account of sampling strategy |  |  |
|  |  | (*e*) Describe any sensitivity analyses |  |  |
| Results | | | | |
| Participants | 13* | (a) Report numbers of individuals at each stage of study—eg numbers potentially eligible, examined for eligibility, confirmed eligible, included in the study, completing follow-up, and analysed | Page 6 | 94 ES-SCLC patients included; 82% mortality during follow-up. |
|  |  | (b) Give reasons for non-participation at each stage |  |  |
|  |  | (c) Consider use of a flow diagram |  |  |
| Descriptive data | 14* | (a) Give characteristics of study participants (eg demographic, clinical, social) and information on exposures and potential confounders | Page 6 | Mean age: 62.6; 90.4% male; bone most common metastasis (59.6%) |
|  |  | (b) Indicate number of participants with missing data for each variable of interest |  |  |
|  |  | (c) *Cohort study*—Summarise follow-up time (eg, average and total amount) |  |  |
| Outcome data | 15* | *Cohort study*—Report numbers of outcome events or summary measures over time |  |  |
|  |  | *Case-control study—*Report numbers in each exposure category, or summary measures of exposure | Page 6 | Mean OS: Low IPI: 23 mo; High IPI: 9 mo (p = 0.005) |
|  |  | *Cross-sectional study—*Report numbers of outcome events or summary measures |  |  |
| Main results | 16 | (*a*) Give unadjusted estimates and, if applicable, confounder-adjusted estimates and their precision (eg, 95% confidence interval). Make clear which confounders were adjusted for and why they were included | Page 7 | High IPI independently associated with poorer OS.  HR (univariate): 1.029; HR (multivariate): 1.081 |
|  |  | (*b*) Report category boundaries when continuous variables were categorized |  |  |
|  |  | (*c*) If relevant, consider translating estimates of relative risk into absolute risk for a meaningful time period |  |  |

Continued on next page

| Other analyses | 17 | Report other analyses done—eg analyses of subgroups and interactions, and sensitivity analyses | Page 6-7 | IPI is an independent prognostic factor in ES-SCLC. |
| --- | --- | --- | --- | --- |
| Discussion | | | | |
| Key results | 18 | Summarise key results with reference to study objectives | Page 7 | Our study found that baseline PNI levels at admission in SAP patients were significantly lower in the in-hospital mortality group. |
| Limitations | 19 | Discuss limitations of the study, taking into account sources of potential bias or imprecision. Discuss both direction and magnitude of any potential bias | Page 10 | Single-center, retrospective design; male-dominated cohort; limited external validity. |
| Interpretation | 20 | Give a cautious overall interpretation of results considering objectives, limitations, multiplicity of analyses, results from similar studies, and other relevant evidence | Page 10 | IPI is a cost-effective, accessible prognostic index. Larger multicenter validation studies needed. |
| Generalisability | 21 | Discuss the generalisability (external validity) of the study results | Page 10 | Findings support using IPI for risk stratification in ES-SCLC, but generalizability limited by sample. |
| Other information | |  | | |
| Funding | 22 | Give the source of funding and the role of the funders for the present study and, if applicable, for the original study on which the present article is based | Page 10 | None |

*Give information separately for cases and controls in case-control studies and, if applicable, for exposed and unexposed groups in cohort and cross-sectional studies.

**Note:** An Explanation and Elaboration article discusses each checklist item and gives methodological background and published examples of transparent reporting. The STROBE checklist is best used in conjunction with this article (freely available on the Web sites of PLoS Medicine at http://www.plosmedicine.org/, Annals of Internal Medicine at http://www.annals.org/, and Epidemiology at http://www.epidem.com/). Information on the STROBE Initiative is available at www.strobe-statement.org.
